# Age-specific prevalence, transmission and phylogeny of TT virus in the Czech Republic

**DOI:** 10.1186/1471-2334-4-56

**Published:** 2004-12-03

**Authors:** Martina Saláková, Vratislav Němeček, Jaroslav König, Ruth Tachezy

**Affiliations:** 1 Department of Experimental Virology, Institute of Hematology and Blood Transfusion, U Nemocnice 1, 128 20 Prague 2, Czech Republic; 2 National Reference Laboratory for Hepatitis, National Institute of Health, Prague, Czech Republic

## Abstract

**Background:**

TT virus is prevalent worldwide, but its prevalence and genotype distribution in Central and East-Europe has not been determined. The high prevalence of TTV in multiply-transfused patients points to the importance of a parenteral mode of transmission, but since more than half of the general population is infected other possible routes of transmission must be considered.

**Methods:**

In our study, we investigated the epidemiology, transmission and phylogeny of TTV in the Czech Republic. The following groups were selected: a control group of 196 blood donors, 20 patients with hemophilia, 49 intravenous drug users, 100 sex workers, 50 penitentiary prisoners, 208 healthy children aged 1 to 14 years, 54 cord blood samples, 52 patients with non-A-E hepatitis, 74 patients with hepatitis C, and 51 blood donors with increased ALT levels. Primers specific for the non-coding region were used. The genotype distribution was studied in 70 TTV-positive samples.

**Results:**

The prevalence rate of TTV among the Czech population was 52.6%. We have shown that TTV is not transmitted prenatally. Children were infected after birth with two peaks: one at the age of two years and the other after the beginning of primary school. Adults have shown a further increase in the TTV prevalence with age. The highest TTV prevalence was found in the group of patients who had received multiple blood transfusions. The TTV prevalence rate in subjects at an increased risk of sexual transmission was not significantly higher than in the general population. Genotypes G2 and G1 were most prevalent among the Czech population, followed by G8 and G3. The subjects positive for markers of HBV and/or HCV infection tested significantly more often TTV DNA positive, which is suggestive of a common route of transmission of these three infections.

**Conclusions:**

This study on TTV prevalence, mode of transmission and age-specific prevalence is the most extensive study performed in Central and Eastern Europe. It showed insights into the epidemiology of TTV infection, but failed to associate TTV infection with clinical manifestations.

## Background

TTV, a non-enveloped small circular single-stranded DNA virus, was recently placed in a novel virus family named Circinoviridae [1]. In spite of being a DNA virus, TTV has an extremely wide range of sequence divergence. At least 40 TTV genotypes from five major phylogenetic groups (G1–G5) have been identified [2,3]. The evolutionary distance between the classified genotypes, as measured by nucleotide substitutions per site, is greater than 30% in the N22 region of ORF1 [4].

TTV has a worldwide distribution. The prevalence of the most common TTV genotypes G1 and G2 is similar all over the world, while the reports on the distribution of other genotypes are scarce and not conclusive. The rate of TTV DNA detection is influenced by the selection of the primer annealing sites. PCR using primers which target the ORF1 region can detect only TTV genotypes 1–6 of group 1, but PCR primers designed for the NCR can detect nearly all genotypes or genetic groups known so far [2].

The prevalence of TT virus in the general population has ranged between 1.9 and 98%, with the highest rates detected in the African and South American countries [5,6]. The TTV prevalence and genotype distribution in Central and East Europe have not yet been determined. One Polish study has reported the prevalence of TTV in blood donors to be 10% by ORF1 primers and 78% by NCR primers [7]. In a small group of blood donors in the Czech Republic, Krekulova et al. detected a TTV prevalence rate of 13.5% by ORF1 primers [8].

The mechanisms of TTV transmission have not yet been elucidated. Even though numerous studies have suggested that the parenteral transmission via transfusion of contaminated blood and blood products is the most common route of TTV infection [9], the detection of TTV in many individuals with no history of blood transfusion indicates that other routes of transmission of TTV may exist. This assumption has been further supported by the detection of TTV in saliva [10], breast milk [11], semen [12] and vaginal fluid [13]. There is evidence that TTV is excreted into feces of infected individuals, suggestive of possible fecal-oral transmission [14]. Some studies have reported placental transmission of TTV [15-17], whereas others have not detected TTV in cord blood and amniotic fluid [18,19]. Since children of TTV-infected mothers apparently tend to get infected more often and earlier after birth than children of TTV negative mothers, the role of postnatal transmission of TTV is being considered [20,21]. Furthermore, variation in the TTV prevalence in children from 5.1% in Japan [22] to 54% in the Democratic Republic of Congo [23] is also suggestive of the possible involvement of other specific environmental factors in the acquisition of TTV infection.

TTV was originally isolated from a blood-transfused patients in which increased alanine aminotransferase (ALT) levels were detected [24]. Therefore, TTV was thought to be the possible etiological factor of non-A-G hepatitis. However, further research has ruled out the notion that a clinically evident liver disease is a consequence of TTV infection. Other studies, which investigated the possible link of TTV to other than hepatic diseases are scarce and so far fail to show any association of TTV infection with clinical manifestation. Nevertheless, the spectrum of diseases studied in association with TTV infection is very narrow and justifies keeping TTV in the category of "orphan" viruses [25].

To investigate the possible routes of TTV transmission, age-specific prevalence and genotype distribution in the population of the Czech Republic, we analyzed sera of 854 subjects divided into 5 groups based on the type of risk of TTV transmission. The age-specific distribution of TTV and correlation with the anti-HBV and/or anti-HCV status were determined. TTV genotypes were determined in 70 patients selected from the different groups.

## Methods

### Population studied

The Human Subjects Committee of the Institutional Review Boards approved all experimental protocols, and all subjects enrolled in the study signed an informed consent form. Additional samples were obtained from the collection of the national reference laboratory for viral hepatitis (NRL-VH). Five groups of subjects were analyzed:

Group 1 (normal population, control group) was selected from 778 healthy blood donors (mean age 29 years, age range 18–59 years). All donors had normal ALT levels, and were negative for anti-HCV, anti-HIV and HBsAg. Since the TTV DNA prevalence in the first 100 subjects tested was very high we decided to randomly select sera [26] to include 20 first-time donors and 10 regular donors from each of five age groups (18–20, 21–30, 31–40, 41–50 and 50–60 years). In total, 136 sera of first-time donors and 60 sera of regular blood donors were analyzed.

Group 2 (at high risk of parenterally transmitted infection) consisted of 20 hemophiliacs (peripheral blood mononuclear cells (PBMCs) were collected from 10 patients) (mean age 42 years, age range 21–74 years) and 49 IVDUs. Patients with hemophilia were screened for anti-HCV, anti-HIV, anti-HBc and HBsAg, IVDUs were tested for anti-HBc and anti-HCV.

Group 3 (at high risk of sexually transmitted infection) included 85 sex workers and 15 promiscuous men (mean age 25 years, age range 18–43 years). All of them were screened for anti-HCV, anti-HBc, HbsAg and HIV.

Fifty penitentiary prisoners (mean age 29, age range 16–58 years) were tested for a potential increased risk of parenteral and/or sexual transmission of the virus. They were screened for anti-HCV and HBsAg.

Group 4 (at risk of transuterine and mother to child virus transmission) consisted of 54 cord blood samples and 208 sera of children selected by age (we analyzed 28–30 subjects in each of the following 7 age groups: 1, 2, 3, 5, 8, 11 and 14-year-olds).

Group 5 (at risk of potential etiological involvement of TTV) included 52 patients with non-A-E hepatitis (mean age 40 years, age range 9–76 years), 51 blood donors with elevated ALT levels (mean age 39 years, age range 25–64 years) and 74 patients with hepatitis C (mean age 27 years, age range 2–56 years). All blood donors with elevated ALT levels and all patients with non-A-E hepatitis tested negative for anti-HCV and HBsAg. All blood donors were also negative for anti-HIV. All patients with hepatitis C were HBsAg negative.

### DNA purification

#### DNA extraction from sera

DNA was extracted from 200 μL of serum using the QIAamp Blood kit (QIAGEN Ltd., Crawley, UK) and dissolved in 100 μL of elution buffer (QIAGEN Ltd., Crawley, UK). Extracted DNA was stored at -20°C.

#### DNA extraction from PBMCs

PBMCs were separated by centrifugation from the whole blood on a Ficoll-Paque gradient (SIGMA, St. Louis, MO) according to the manufacturer's protocol. PBMCs were digested with proteinase K (100 μg/ml) (SIGMA, St. Louis, MO) in 1 ml of lysis buffer (50 mM Tris-HCl, pH 8.0; 1 % Tween; 5 mM EDTA, pH 8.0). Thereafter, proteinase K was inactivated for 10 min at 95°C and samples were stored at -20°C.

### Polymerase chain reaction

Five microlitres of total DNA were analyzed in a PCR thermocycler PTC 200 (MJ Research, Inc, Waltham, MA). TTV DNA detection was performed with two sets of primers. A 271 bp long fragment of the ORF1 was amplified in a semi-nested PCR with a modified primer set designed by Okamoto [4]. By nested PCR a 110 bp fragment was amplified from the NCR [2]. For the first PCR with ORF1 specific primers, 50 pmol of both modified primers NG059mod (3' CAGACAGAGGMGAAGGMAAYATG 5') and NG063mod (3'CTGGCATYTYWCC MTTTCCAAARTT 5') were used in a 50 μl reaction mixture containing 10 mM Tris-HCl, pH 8.8, 50 mM KCl, 0.8% Nonidet P40, 200 μM each dNTP and 2.5 U Taq polymerase (Fermentas, Hanover, MD). Each of the 35 cycles consisted of 30 s of denaturation at 94°C, 30 s of annealing at 58°C and 45 s of elongation at 72°C. The last cycle was followed by 7 min incubation at 72°C. One microliter of the product from the first PCR was transferred to 50 μl reaction mixture with primers NG061 (3' GGMAAYATGYTRTGGATAGACTGG 5') and NG063mod. The reaction mixture and cycling conditions for the second PCR were the same except that 25 cycles were run.

For the PCR with NCR specific primers the same conditions as for ORF1 specific PCR were used. In the first PCR we used 50 pmol of primer NG133 (3'GTAAGTGCACTTCCGAATGGCTGAG 5') and NG147 (3'GCCAGTCCCGAGCCCG AATTGCC 5'), for the second PCR 50 pmol of primers NG134 (3'AGTTTTCCA CGCCCGTCCGCAGC 5') and NG132 (3'AGCCCGAATTGCCCCTTG AC 5'). Each of the 35 cycles of the first PCR and 25 cycles of the second PCR consisted of 30 s of denaturation at 94°C, 30 s of annealing at 58°C and 45 s of elongation at 72°C, with a final extension for 7 min at 72°C. Ten microlitres of the PCR product were separated electrophoretically on a 3% agarose gel (NuSieve 3:1, FMC BioProduct, Rockland, ME).

### TTV viral load in serum and PBMCs of patients with hemophilia

DNA extracted from sera and PBMCs of 4 patients with hemophilia was serially diluted (in 10-fold steps) in distilled water and TTV DNA was determined by PCR with NCR primers. The highest dilution (10^N^) testing positive was used as a relative titer for determining the viral titer per 1 ml of TTV DNA in serum and PBMCs.

### Sequence analysis

A nucleotide DNA sequence of PCR ORF1 products (selected from all groups of study subjects), which revealed a clear band on the agarose gel, was determined. PCR products were excised from a 3% agarose gel and purified with MinElute Gel Extraction kit (QIAGEN Ltd., Crawley, UK) according to the manufacturer's protocol. Both strands of the 271 bp long products generated by PCR with ORF1-specific primers were sequenced directly with the NG061 and NG063mod primers using the ABI Big Dye Sequencing kit (Applied Biosystems, Foster City, CA). The sequencing was performed on an ABI PRISM 310 automated DNA sequencer (Applied Biosystems, Foster City, CA).

### Phylogenetic analysis

DNA sequences (of the following accession numbers: AY429576 – AY429589, AY433961 – AY434008, AY456097 – AY456103, AY484597) were aligned using the CLUSTAL X program [27] with the corresponding 222 bp long ORF1 region of previously reported sequences (of the following accessions numbers: AB017768 – AB017770, AB017774 – AB017779, AB017886, AB018889, AB018961, AB021796, AB021798, AB021800, AB021803, AB021815, AF060546, AF060547, AF072749, AF077274, AF079541, AF123914, AF123948, AF124009, AF124027) obtained from GenBank at NCBI (NCBI, Bethesda, MA). Phylogenetic trees were constructed using the neighbor-joining and maximum likelihood method in PHYLIP package, version 3.5 [28].

### Serology of hepatitis B and C

HBsAg (V2) AxSYM, CORE AxSYM, AUSAB AxSYM, HCV3.0 AxSYM (Abbott, Chicago, IL) tests were used for the detection of HBsAg, anti-HBc, anti-HBs and anti-HCV markers. HBsAg reactive samples were confirmed with the HBsAg Confirmatory AxSYM test.

### Statistical analysis

The statistical analysis was performed using the Fisher exact test. Odds ratios (OR) with 95% confidence intervals (CI) and two-tailed P values were calculated in 2 – 2 tables using the EPI INFO statistical package (version 2002) and GraphPad InStat (version 3.05) (GraphPad Software, San Diego, CA). In all tests, the basic significance level was P = 0.05.

## Results

In total, 854 samples were screened for the presence of TTV DNA using a NCR-PCR. The TTV genotype was determined by sequencing part of the ORF1 region from 70 TTV isolates.

### Prevalence of TTV DNA in different groups of subjects

The prevalence rates of TTV DNA in different groups are shown in Table 1. The prevalence of TTV DNA in healthy blood donors representing the normal population was 52.6% (103/196). We found no difference in the prevalence of TTV DNA between first-time and regular blood donors (results not shown). The highest prevalence rates were recorded in group 2 (at higher risk of parenteral transmission of infection): 95% (19/20) (OR = 17.16, CI 2.25–130.73, P = 0.0002) for hemophiliacs and 91.8% (45/49) (OR = 10.16, CI 3.52–29.34, P < 0.0001) for IVDUs. In group 3, sex workers and promiscuous men had a prevalence of TTV DNA comparable with that of the normal population, i.e. 62% (62/100), but penitentiary prisoners had a significantly higher prevalence of TTV DNA, i.e. 74% (37/50) (OR = 2.38, CI 1.21–4.69, P = 0.011) than blood donors. We detected no TTV DNA in the cord blood samples, but children had a similar prevalence of TTV DNA as the control group, i.e. 67.8% (141/208). Patients in group 5 had a slightly higher prevalence of TTV DNA than healthy blood donors, the difference being significant only for patients with hepatitis C virus infection, i.e. 89.2% (66/74) (OR = 7.45, CI 3.40–16.3, P < 0.0001).

### Age-specific prevalence of TTV DNA

**Figure 1:**
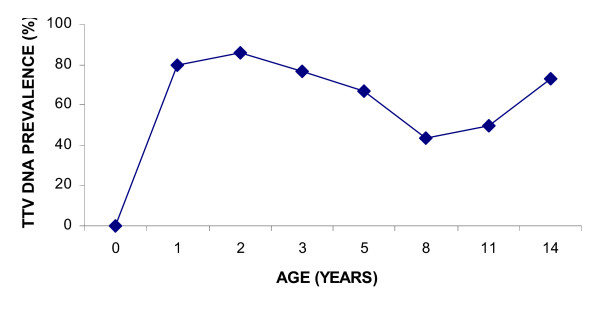
Age specific prevalence of TTV in children Age-dependent prevalence of TTV DNA in children

**Figure 2:**
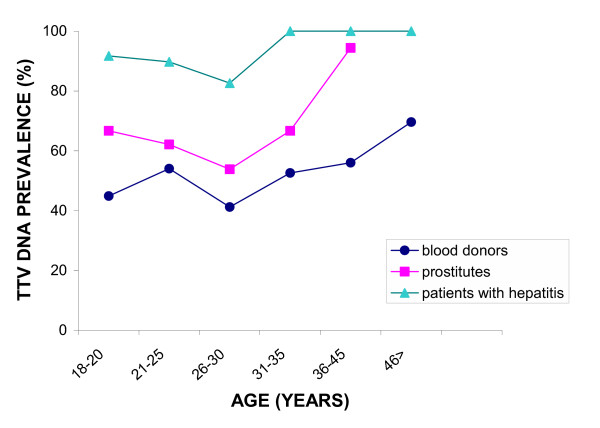
Age specific prevalence of TTV in adults Age-specific prevalence of TTV DNA in the control group, in the group at increased risk of sexually transmitted infection and in patients with non-A-E hepatitis or hepatitis C as detected with NCR-specific primers

As indicated in Figure 1, we observed a dramatic increase in TTV-prevalence during the first two years of life (at the age of 2 years it equaled 85.7%). The prevalence decreased to 43.3% for 8-year-olds and started to rise again to 73.3% in 14-year-olds. Figure 2 shows an age-dependent distribution pattern of TTV DNA prevalence in the three groups of subjects (blood donors, a group at increased risk of sexual infection, and a combined group of patients with non-A-E hepatitis and hepatitis C). TTV prevalence showed a tendency to increase with a significant linear trend for the group at a higher risk of sexual transmission (Chi square for trend= 8.002, p = 0.0047). Even although the control group showed an obvious increase in the TTV DNA prevalence with age, this trend was not significant.

### TTV presence and viral load in serum and PBMCs

As indicated in Table 2 for patients with hemophilia, TTV DNA was more often detected in serum than in PBMCs. TTV DNA was found in sera of all patients (10/10, 100%) and in most (7/10, 70%) PBMCs. Seven patients were positive in serum and PBMCs samples, three subjects had TTV DNA detectable only in serum. The amplification of the internal control beta-globin gene was positive for all PBMCs samples. Viral loads in PBMCs and in corresponding serum samples were compared for four hemophiliacs. All individuals had TTV DNA titers 10 to 100 times higher in their sera than in PBMCs.

### Heterogeneity of TTV genotypes

**Figure 3:**
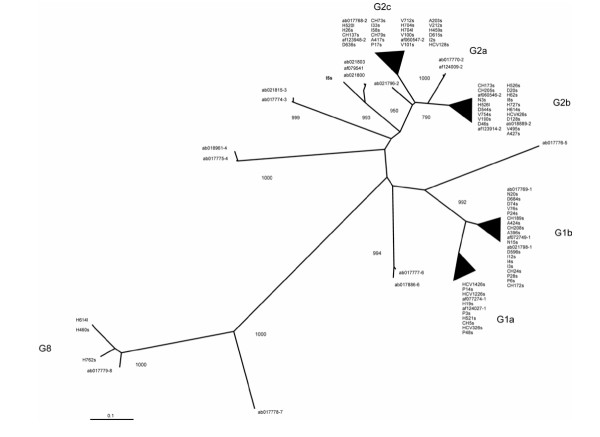
Phylogeny analysis of the Czech TTV isolates Phylogenetic tree constructed by neibor-joining method from a 222 bp fragment of the ORF1 region obtained by sequencing 70 isolates from the Czech population (accession numbers: AY429576 – AY429589, AY433961 – AY434008, AY456097 – AY456103, AY484597) and from previously reported sequences (accessions numbers: AB017768 – AB017770, AB017774 – AB017779, AB017886, AB018889, AB018961, AB021796, AB021798, AB021800, AB021803, AB021815, AF060546, AF060547, AF072749, AF077274, AF079541, AF123914, AF123948, AF124009, AF124027). The groups in which the isolates from our study were situated are circled and the I5s isolate which is located on a separate branch is in bold. Bootstrap values above 75% are shown.

The results of our sequencing and phylogenetic analysis are summarized in Table 3 and Figure 3. The topography of the tree using either the neighbour-joining or maximum likelihood method was identical. We analysed 9 samples from group 1, 23 from group 2, 15 from group 3, 10 from group 4 and 13 from group 5. According to Okamoto's classification [2], 9 isolates were classified into genotype G1a (2 patients with hemophilia, 3 penitentiary prisoners, 1 child, 3 patients with hepatitis C), 18 isolates into genotype G1b (3 blood donors, 3 IVDUs, 1 prostitute, 3 penitentiary prisoners, 4 children, 2 blood donors with increased ALT levels and 2 patients with non-A-E hepatitis), 18 isolates into genotype G2b (4 blood donors, 5 patients with hemophilia, 1 IVDU, 3 sex workers, 2 children, 1 blood donors with an elevated ALT level, 1 patient with hepatitis C and 1 patient with non-A-E hepatitis), 21 isolates into genotype G2c (2 blood donors, 5 patients with hemophilia, 3 IVDUs, 4 sex workers, 1 penitentiary prisoner, 3 children, 2 blood donors with increased ALT levels, 1 patient with hepatitis C), 3 isolates into genotype G8 (3 patients with hemophilia) (Table 3) and 1 isolate from a IVDU (I5s) was most closely related to genotype G2. The similarity of this product with a G2a reference genotype sequence (AB017770) was 74.8 %; its similarity with the reference sequence of G2c (AB017768) was 73.9% on the nucleotide level. In the Czech population, the most prevalent genotype was G2c (30.0%), followed by G1b (25.7%), G2b (25.7%), G1a (12.9%) and G8 (4.3%). No association between any of the detected genotypes and a particular population group was revealed.

### Comparison of TTV genotypes present in sera and PBMCs of the same patient

Tree of four patients positive in both serum and PBMCs by the ORF1 PCR system yielded a PCR product adequate for sequencing analysis. Genotypes and sequence homologies are shown in Table 2. Two paired sequences were of the same genotype, G2b and G2c, respectively, and the sequence similarity between the genotype detected in PBMCs and serum was 96.8% and 100%, respectively. In one patient we detected genotype G8 in PBMCs and genotype G2b in serum (similarity 32.6%).

### Co-infection markers in association with TTV DNA presence

Four groups of subjects were compared for correlation of past HBV and/or HCV infection with TTV DNA prevalence. We compared subjects at a higher risk of sexual transmission, IVDUs, patients with hemophilia and penitentiary prisoners. There was no evidence of present or past HCV or present HBV infection in blood donors. The prevalence of anti-HBc in blood donors in the Czech Republic is very low (1–2%, unpublished data). These data imply that our control group was at a low risk of sexually and parenterally acquired infections. Also all children were anti-HBc and anti-HCV negative. Both the groups of hemophiliacs and IVDUs presented evidence of frequent past or current HBV (50% and 22%) and HCV (82% and 72%) infection. Thirteen percent of sex workers and promiscuous men showed past or current HBV exposure. The study subjects positive for HBV and/or HCV markers had a significantly higher prevalence of TTV DNA regardless of the primer set used (Table 4).

## Discussion

The present study on TTV prevalence, mode of transmission and age-specific prevalence is the largest study performed in Central and East Europe. This study showed that TTV infection is quite common among the Czech population. The prevalence rates of TTV in blood donors (52.6%) were similar to those found in other developed countries (for review see [29]).

The most interesting result was the lack of TTV DNA in 54 cord blood samples, suggestive of the absence of transuterine transmission of TTV. Furthermore, we have shown that the prevalence of TTV DNA was age dependent. In children, we observed a dramatic increase of TTV prevalence within the first two years of age. The prevalence of TTV further gradually increased in children aged 8 to 14 years. Similarly, Ohto et al. reported that the TTV prevalence in children at the age of 2 years was comparable with that in mothers, while children younger than 3 months of age were infected only exceptionally [19]. In the Czech Republic, children start schooling at the age of six or seven years. Based on our data, postnatal infection from mother to child and an increased number of social contacts are likely to be the most important routes of TTV transmission in children.

In adults, we have shown the increase of TTV prevalence with age irrespective of study group. Similar results have been reported by others [21,30-33].

We observed an increased prevalence of TTV in various groups of hepatitis patients. Since many hepatitis viruses share the same modes of transmission, multiple viral infections may occur in one patient [34]. Our results showed a significantly higher prevalence of TTV infection in patients with hepatitis C than in healthy individuals, implying that HCV and TTV may share common modes of transmission.

In agreement with other studies we detected the highest prevalence of TTV in hemophiliacs and IVDUs, which supports the importance of the parenteral route of transmission of TTV [9,35-38]. Nevertheless, the prevalence of 52.6% in blood donors and in healthy children suggests that the higher prevalence in hemophiliacs and IVDUs can also be attributed to a higher TTV viral load. Different TTV concentrations of virus in hemophiliacs and blood donors have been previously reported by Touinssi [39]. Additionally, Simmonds has shown that in hemophiliacs the prevalence of TTV increased with the amount of clotting factor treatment received and was also dependent on whether the blood concentrates tested had been virally inactivated [9].

As for TTV sexual transmission, the results of our study suggest that if TTV is sexually transmitted, this mode of transmission is likely to be less important. Even though 13% subjects of the group at risk for sexual transmission of TTV showed past or current HBV exposure, indicative of high promiscuity, the prevalence of TTV was not significantly different from that of the control group (62% and 52.6%). To the best of our knowledge, so far only one study found a significant difference in the TTV prevalence in sex workers [40], while others did not.

In the group of penitentiary prisoners the significantly higher prevalence of TTV seems to be most probably a consequence of intravenous drug abuse rather than sexual promiscuity since the prevalence of HCV was four-times higher than in the group at risk for sexual transmission (20% versus 5%).

An increased prevalence of TTV in non-A-E hepatitis patients was observed in the present study in agreement with many previous studies. Additionally, our data showed an increased TTV prevalence in blood donors with increased ALT levels. Several studies have shown a correlation between TTV-titer and elevation of serum ALT levels [24,30] but the experimental infection of chimpanzees with TTV did not show any biochemical or histological evidence of hepatitis [1].

The heterogeneity of TTV is extreme. Because the NCR region is too conserved for evolutionary analyses, the ORF1 region of the TTV genome is most often used for genotyping. Out of the 70 sequenced isolates, G2 followed by G1 were the most frequent genotypes among the Czech population. The phylogeny analysis showed no evidence of association of particular TTV genotypes with any of the risk groups.

## Conclusions

Our results demonstrated a high prevalence of TTV in the Czech population. Our data show the absence of transuterine transmission of TTV, but postnatal route of transmission from mother to child and infection via frequent social contacts seem to be very important modes of transmision in children. The sexual mode of transmission is most likely to be low effective. No convincing evidence was found to support the involvement of TTV in the pathogenesis of hepatitis.

Our data, as well as the results of other studies, show that optimization of the primer set for more standard TTV detection and genotyping is still needed. Improved serological approach to TTV detection could be of value. It is evident that more data are still needed for a better understanding of the natural history of TTV infection.

## Competing interests

The author(s) declare that they have no competing interests.

## Authors'contributions

RT and VN conceived of the study and participated in its design and coordination. MS carried out most of the experimental work and participated on the preparation of the initial draft of the manuscript. Part of the experimental work was done by JK. RT did all the statistical analysis and evaluation of the results and writing of the final version of the manuscript. All authors contributed to the preparation of the final manuscript.

## Pre-publication history

The pre-publication history for this paper can be accessed here:



## Figures and Tables

**Table 1:** Prevalence of TTV DNA by study groups

Number	Study group	Number of subjects	Number of TTV DNA positives (%)	Difference (p value)
1	blood donors	196	103 (52.6%)	
2	patients with hemophilia	20	19 (95.0%)	<0.001
2	IDVUs	49	45 (91.8%)	<0.0001
3	sex workers	100	62 (62.0%)	NS
2, 3	penitentiary prisoners	50	37 (74.0%)	<0.05
4	children	208	141 (67.8%)	<0.005
4	cord blood samples	54	0 (0%)	-
5	patients with non A-E hepatitis	52	39 (75.0%)	<0.005
5	patients with HCV	74	66 (89.2%)	<0.0001
5	blood donors with increased ALT levels	51	31 (60.8%)	NS

Study groups: 1 = controls, 2 = subjects at high risk of parenterally transmitted infection, 3 = subjects at high risk of sexually transmitted infection, 4 = subjects at risk of transuterine or mother to child transmitted infection, 5 = subjects at risk of potential etiological involvement of TTV

**Table 2:** Comparison of TTV viral loads in sera and PBMCs of patients with hemophilia

Sample	Type of material	Genotype	Similarity of genotypes (%)	Viral load (relative titer 10^N^/ml)
1	serum	G2c	100	10^7^
	PBMCs	G2c		10^6^
2	serum	G2b	96.8	10^7^
	PBMCs	G2b		10^6^
3	serum	G2b	32.6	10^7^
	PBMCs	G8		10^6^
4	serum	-	-	10^7^
	PBMCs	G2c		10^5^

**Table 3:** TTV GENOTYPES BY STUDY GROUPS

			TTV genotype					
Group	Number of positive subjects	Number of sequenced samples	G1a	G1b	G2b	G2c	G8	other

blood donors	14	9	-	3	4	2	-	-
patients with hemophilia	14	15^a^	2	-	5^b^	5^c^	3^d^	-
IDVUs	14	8	-	3	1	3	-	1
sex workers	10	8	-	1	3	4	-	-
penitentiary prisoners	10	7	3	3	-	1	-	-
children	17	10	1	4	2	3	-	-
cord blood samples	0	0	-	-	-	-	-	-
patients with non A-E hepatitis	7	3	-	2	1	-	-	-
patients with HCV	14	5	3	-	1	1	-	-
blood donors with increased ALT levels	8	5	0	2	1	2	-	-
Total (%)	108	70	9 (12.9)	18 (25.7)	18 (25.7)	21 (30.0)	3 (4.3)	1 (1.4)

a) 11 PCR products amplified from sera and 4 PCR products amplified from DNA extracted from PBMCs

b) 1 PBMC sample positive for G2b

c) 2 PBMC samples positive for G2c

d) 1 PBMC sample positive for G8

**Table 4:** TTV presence in relation to the past infection with HBV and/or HCV

HBV and HCV serology		TTV positive subjects			
		
		PCR	OR	CI	P
anti-HBc	positive	39/42 (92.9%)	5.452	1.61–18.44	<0.01
	negative	124/176 (70.5%)			
Anti-HCV	positive	54/59 (91.5%)	4.954	1.87–13.14	0.0004
	negative	109/159 (68.6%)			
